# Effects and Molecular Mechanism of Single-Nucleotide Polymorphisms of MEG3 on Porcine Skeletal Muscle Development

**DOI:** 10.3389/fgene.2021.607910

**Published:** 2021-02-22

**Authors:** Rui Yang, Yinuo Liu, Yunyun Cheng, Chunli Wang, Jie Song, Guanhong Lu, Tianqi Feng, Siyao Wang, Xiaotong Sun, Jilun Meng, Linlin Hao

**Affiliations:** ^1^College of Animal Science, Jilin University, Changchun, China; ^2^Zhejiang Institute of Freshwater Fisheries, Huzhou, China; ^3^College of Public Health, Jilin University, Changchun, China

**Keywords:** long non-coding RNA, skeletal muscle, SNPs, pig, MEG3

## Abstract

Maternally expressed gene 3 (MEG3) is a long non-coding RNA that is a crucial regulator of skeletal muscle development. Some single-nucleotide polymorphism (SNP) mutants in MEG3 had strong associations with meat quality traits. Nevertheless, the function and mechanism of MEG3 mutants on porcine skeletal muscle development have not yet been well-demonstrated. In this study, eight SNPs were identified in MEG3 of fat- and lean-type pig breeds. Four of these SNPs (g.3087C > T, g.3108C > T, g.3398C > T, and g.3971A > C) were significantly associated with meat quality and consisted of the CCCA haplotype for fat-type pigs and the TTCC haplotype for lean-type pigs. Quantitative real-time PCR results showed that the expression of MEG3-TTCC was higher than that of MEG3-CCCA in transcription level (*P* < 0.01). The stability assay showed that the lncRNA stability of MEG3-TTCC was lower than that of MEG3-CCCA (*P* < 0.05). Furthermore, the results of qRT-PCR, Western blot, and Cell Counting Kit-8 assays demonstrated that the overexpression of MEG3-TTCC more significantly inhibited the proliferation of porcine skeletal muscle satellite cells (SCs) than that of MEG3-CCCA (*P* < 0.05). Moreover, the overexpression of MEG3-TTCC more significantly promoted the differentiation of SCs than that of MEG3-CCCA (*P* < 0.05). The Western blot assay suggested that the overexpression of MEG3-TTCC and MEG3-CCCA inhibited the proliferation of SCs by inhibiting PI3K/AKT and MAPK/ERK1/2 signaling pathways. The overexpression of the two haplotypes also promoted the differentiation of SCs by activating the JAK2/STAT3 signaling pathway in different degrees. These data are valuable for further studies on understanding the crucial role of lncRNAs in skeletal muscle development.

## Introduction

Skeletal muscle is the most abundant muscle in mammals (Ge et al., 2019; Han et al., 2019). Skeletal muscle satellite cells (SCs) are located between the myofiber membrane and basal lamina membrane of muscle fibers (Koning et al., 2012; Guo et al., 2018). SCs are terminally differentiated into multinucleated myotubes and myofibers, which are essential for postnatal growth and regeneration of skeletal muscles (Felicioni et al., 2020). The meat quality of different pig breeds is associated with proliferation and differentiation of SCs (Gao et al., 2017). BaMa Xiang pig (BaMa) is a distinguished Chinese indigenous pig breed with a fat genotype and always is called as fat-type pig in some studies (Liu et al., 2010, 2015; Yang et al., 2014). In contrast, European commercial pig breeds are typically lean breeds with representative lean genotype and are also widely used as the experimental objects (Gil et al., 2008; Wu et al., 2013; Yang et al., 2014), such as Landrace pigs, Duroc pigs, Yorkshire pigs, and Pietrain pigs have faster growth rate and higher lean meat content than BaMa pig breeds (Liu et al., 2015; Li et al., 2016). In addition, according to the Chinese Livestock or Poultry Genetic Resource-Pigs (National Committee for Livestock or Poultry Genetic Resource, 2011), the lean meat content (%) of European commercial pig breeds is higher than that of BaMa pig breed (*P* < 0.05).

Long non-coding RNAs (lncRNAs) refer to transcripts exceeding 200 nucleotides without protein-coding ability (Jarroux et al., 2017). The regulatory role of lncRNAs in skeletal muscle growth has emerged in recent years. For instance, MAR1 is a lncRNA that promotes skeletal muscle differentiation by regulating myogenic mediator Wnt5a (Zhang et al., 2018). Gong et al. (2015) demonstrated that lncMyoD regulated skeletal muscle differentiation by blocking IMP2-mediated mRNA translation. MEG3 (Maternally expressed gene 3), a lncRNA identified first in mice, was reported to be a regulator of skeletal muscle development (Yu et al., 2018). A previous study demonstrated that MEG3 promoted bovine skeletal muscle differentiation by interacting with miRNA-135 and MEF2C (Chen et al., 2013). Zhou et al. (2010) illustrated that maternal deletion of MEG3 resulted in perinatal death and skeletal muscle defects. MEG3 was expressed in eight different tissues including heart, liver, spleen, lung, kidney, intestine, stomach, and skeletal muscle in Large White pigs (Yu et al., 2018). The MEG3 expression was higher in skeletal muscle than in other tissues (Yu et al., 2018). Furthermore, previous researches suggested that MEG3 overexpression caused a decrease of cell proliferation and downregulations of proteins involved in proliferation, including CDK4, CDK6, and CyclinD1 (Ji and Li, 2019). Meanwhile, many studies demonstrated that MEG3 could affect myogenic differentiation of skeletal muscle cell by regulation of the myogenic marker, MyoD, MyoG, and MyHC (Zhang et al., 2018). These studies demonstrated that MEG3 may play a role in molecular mechanisms of skeletal muscle development.

Single-nucleotide polymorphisms (SNPs) represent the most frequent genetic changes in the mammal genome and may affect the RNA secondary structure and expression level of lncRNAs (Chen et al., 2013; Minotti et al., 2018). For example, an SNP site (rs1015164), which is located in the first intron of lncRNA CCR5AS, altered the expression of lncRNA CCR5AS and finally regulated the expression and function of co-receptor CCR5 (Kulkarni et al., 2019). The SNP site (rs1859168) located in the HOTTIP (HOXA transcript at the distal tip), a lncRNA transcribed from the 5′ tip of the HOXA locus, influenced the RNA secondary structure by decreasing free energy, which consequently affected HOTTIP expression and function (Minotti et al., 2018). Increasing lines of evidence indicate the important role of SNPs in lncRNAs associated with some phenotypes, such as disease (Zheng et al., 2020), muscle weight (Zhou et al., 2018), muscle fiber width, and muscle fiber roundness (Ren et al., 2017). However, the potential effects of SNPs located in lncRNAs on the growth and development of porcine remain unexplored. The influences of SNPs located in MEG3 on porcine skeletal muscle development and the underlying potential molecular mechanisms remain unknown. This study aimed to investigate whether SNPs located in MEG3 will affect the transcription level of MEG3 and influence the proliferation and differentiation of porcine SCs.

## Materials and Methods

### Animals

Animal experimental procedures were approved by the Institutional Animal Care and Use Committee of Jilin University (Changchun, China, Approval number SY201912002, 2 December 2019). All animal welfare and experimental procedures were performed strictly according to the guidelines from the National Institutes of Health Guide for the Care and Use of Laboratory Animals (NIH Publications No. 8023, revised 1978).

### Grouping the Animal Samples, DNA Preparation, and SNP Screening of MEG3

With the support of the classification of other studies (Gil et al., 2008; Liu et al., 2010, 2015; Yang et al., 2014) and the characteristics of these breeds provided by Chinese Livestock or Poultry Genetic Resource-Pigs (National Committee for Livestock or Poultry Genetic Resource, 2011, Supplementary Table S1), the Chinese indigenous pig breed (BaMa Pigs) and European pig breeds (Yorkshire, Duroc, Landrace and Pietrain pigs) used in this study were relatively classified into fat-type pig group and lean-type pig group, respectively. Ear notch samples were collected from 22 Yorkshire pigs, 23 Duroc pigs, 20 Landrace pigs, 20 Pietrain pigs, and 75 BaMa pigs, among which the number of female and male pigs was almost equal in each breed. All individuals were selected randomly. No biological relation of each individual was guaranteed for at least three generations within the selected samples. Genomic DNA was extracted using Multisource Genomic DNA Extraction Kit (AXYGEN, China) following the manufacturer’s instructions. Two pairs of primers (MEG3-Exon1 and MEG3-Exon2, Supplementary Table S2) were used to amplify the Exon1 and Exon2 of the MEG3 (NR_021488.1) sequence. PCR amplification was carried out in a total volume of 25 μL of PrimeSTAR HS (Premix) (Takara) with 100 ng of DNA template and 20 pmol of each primer. The PCR conditions included initial heating at 95°C for 5 min, 30 cycles of 30 s for denaturation at 95°C, 30 s for annealing at 58°C, and 30 s for extension at 72°C, followed by a 5 min extension at 72°C. The PCR products were sequenced by Genewiz (Genewiz, China). Geneious software (Biomatters Ltd., United States) was used to analyze all sequences. All primers used were designed by Primer Premier 5.0 software and synthesized by Sangon Biotechnology Company Limited (Shanghai, China).

### Haplotype Analysis

Haplotype test results were analyzed with Haploview v.4.2 (Daly Lab at the Broad Institute Cambridge, United States) (The download address: https://www.broad.mit.edu/mpg/haploview) (Barrett et al., 2005).

### Construction of the Expression Vectors of MEG3 Haplotypes

**FIGURE 1:**
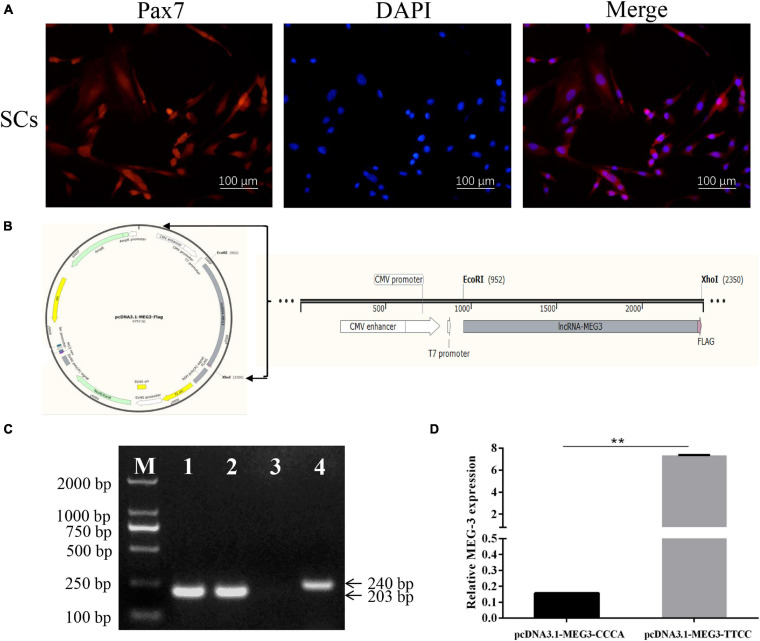
Identification of SCs, construction of the expression vector and the effect of two haplotypes on MEG3 transcription. **(A)** SCs were identified by immunolabeling with anti-Pax7 (red) monolyclonal antibody; Nuclear are counterstained with DAPI (blue); Merged images represented overlays of Pax7 (red) and nuclear staining by DAPI (blue). **(B)** A FLAG tag sequence (pink) was added at the 3′ end of the MEG3 sequence (gray). This fragment was inserted into the pcDNA3.1(+) vector between the EcoR I and Xho I site. **(C)** The RT-PCR results of SCs transfected with pcDNA3.1-MEG3-CCCA, pcDNA3.1-MEG3-TTCC and pcDNA 3.1 (+) vector for 48 h. M: Marker 2000, Line 1–3: the RT-PCR results of pcDNA3.1-MEG3-CCCA group, pcDNA3.1-MEG3-TTCC group and pcDNA 3.1(+) group were amplified by MEG3-FLAG, respectively, Line 4: the RT-PCR results of pcDNA 3.1(+) group were amplified by Primer-GAPDH. **(D)** The effect of two haplotypes on MEG3 transcription. The significance test: ^∗∗^*P* < 0.01.

Full-length MEG3 sequences of lean-type pigs (MEG3-TTCC) and fat-type pigs (MEG3-CCCA) with a FLAG tag sequence (5′-3′: CTTGTCATCGTCGTCCTTGTAGTC) at the 3′ end of MEG3 (Figure 1B) were synthesized in Genewiz (Genewiz, China). The sequences of MEG3-CCCA and MEG3-TTCC were subcloned into the EcoRI and XhoI sites of the pcDNA3.1 (+) vector (Invitrogen, Carlsbad, CA). pcDNA3.1-MEG3-CCCA and pcDNA3.1-MEG3-TTCC were successfully constructed.

### Cell Isolation and Culture

Porcine skeletal muscle satellite cells (SCs) were isolated and cultured following the method described by Wang et al. (2016). Briefly, gastrocnemius muscle tissue from 7-day-old Landrace pig was digested by 0.2% collagenase Type II (Sigma, St. Louis, MO, United States) to release SCs. Differential adhesion method described by Gharaibeh et al. (2008) was used to purify pig SCs. SCs were cultured in growth medium (GM) containing DMEM/F-12 (Gibco, Grand Island, NY, United States), 20% fetal bovine serum (FBS; Gibco), and 1% penicillin and streptomycin (Gibco). The cells were kept in a 37°C incubator with 5% CO_2_. The isolated SCs were seeded in 24-well plates and identified by immunofluorescence staining with anti-mouse Paired box protein Pax7 monoclonal antibody (Santa Cruz Biotechnology, Inc., Europe). The cells were cultured in differentiation medium (DM) containing DMEM/F-12, 5% horse serum (Gibco), and 1% penicillin and streptomycin to induce cell differentiation. The cell proliferation or differentiation medium was changed every 24 h.

### Cell Transfection

SCs were seeded on six-well plates. After the cell confluence reached 30% (for proliferation assay) or 60–70% (for differentiation detection), SCs were transfected with 2 μg of pcDNA3.1-MEG3-CCCA, 2 μg of pcDNA3.1-MEG3-TTCC, and 2 μg of pcDNA3.1 (+) vector with Lipofectamine 2000 (Invitrogen, Carlsbad, CA, United States) according to the manufacturer’s instructions. Cells were harvested after 48 h of culture in GM for proliferation assay or 24 h in GM and 72 h in DM for differentiation detection.

### Cell Proliferation Assay

Cell proliferation was measured at 0, 24, 48, 72, and 96 h by Cell Counting Kit-8 (CCK-8) assay according to the manufacturer’s instructions. In brief, 100 μL of GM supplemented with 10 μL of CCK-8 reagent (Dojindo Molecular Technologies, Inc., Kumamoto, Japan) was added to each well and incubated for another 1 h at 37°C. Absorbance of each sample was recorded at 450 nm by a 96-well microplate reader (TECAN).

### Quantitative Real-Time PCR Assay

Total RNA was extracted using Trizol (Invitrogen Corp, Carlsbad, CA) following the manufacturer’s protocol. RNA (2.5 μg) was used for cDNA synthesis with Revert Aid First-strand cDNA Synthesis Kit (Thermo Fisher Scientific). Quantitative real-time PCR (qRT-PCR) was performed on ABI PRISM 7900HT thermocycler (Applied Biosystems, United States) in triplicate for each sample. The reaction was carried out in a total volume of 20 μL of the qRT-PCR reaction mixture containing 10 μL 2 × SYBR Green PCR Master Mix, 50 ng of cDNA, and 10 pmol of each primer. The qRT-PCR reaction conditions were as follows: denaturation at 95°C for 5 min, followed by 40 cycles of 95°C for 30 s, 58°C for 30 s, and 72°C for 30 s. The expression levels of the target genes were normalized to the GAPDH gene. Data analysis was performed using comparative Ct (2^–ΔΔ*Ct*^) method. The six pairs of primers (Primer-GAPDH, CDK4, CDK6, CyclinD1, MyoG, MyoD) for qRT-PCR are shown in Supplementary Table S2.

### Immunoblot Assay

SCs were washed three times with ice-cold PBS and incubated on ice with lysis buffer (KeyGEN BioTECH, Jiangsu, China) containing Protease Inhibitor Cocktail I (MedChem Express) and Phosphatase Inhibitor Cocktail I (MedChem Express). The mixture was centrifuged at 1,000 × g for 15 min at 4°C. Total protein content was determined by BCA Protein Assay Kit (Beyotime, Shanghai, China). About 30 μg of protein of each sample was loaded onto 10% SDS-polyacrylamide gels and transferred to a polyvinylidene fluoride membrane (Millipore, Billerica, MA, United States). The blots were incubated at 4°C overnight with primary antibodies specific to anti-CDK4 (1:1,000, Wanleibio Co., Ltd., China), anti-CDK6 (1:1,000, Wanleibio Co Ltd, China), anti-CyclinD1 (1:1,000, Wanleibio Co Ltd, China), anti-MyoD (1:1,000, Proteintech, Europe), anti-MyoG (1:1,000, ImmunoWay, United States), anti-p-JAK2 (1:1,000, ImmunoWay, United States), anti-JAK2 (1:1,000, Proteintech, Europe), anti-p-STAT3 (1:1,000, ImmunoWay, United States), anti-STAT3 (1:1,000, Proteintech, Europe), anti-p-AKT (1:1,000, CST, United States), anti-AKT (1:1,000, CST, United States), anti-p-ERK1/2 (1:1,000, CST, United States), anti-ERK1/2 (1:1,000, CST, United States), and GAPDH (1:1,000, BBI, Canada). After being washed with TBST three times, the membranes were incubated by horseradish peroxidase-conjugated anti-Mouse IgG (1:5,000, BBI, Canada) or anti-Rabbit IgG (1:5,000, BBI, Canada) for 1 h at room temperature. The Western blot was visualized by Enhanced Chemiluminescence (ECL, Pharmacia Biotech, Arlington, United States) performed on Bio-Rad Gel Doc XR instrument (Bio-Rad, Hercules, CA, United States). Chemiluminescence was quantified using ImageJ software (National Institutes of Health).

### LncRNA Stability Assay and Prediction of LncRNA Secondary Structures

For lncRNA stability assay, SCs were cultured in six-well plates until 30–40% confluence and then transfected with pcDNA3.1-MEG3-CCCA or pcDNA3.1-MEG3-TTCC. At 24 h after transfection, actinomycin D (ActD; 20 μg/mL) was added to the culture media to block RNA transcription. After treatment, the cells were incubated at 37°C in 5% CO_2_. At selected time points (0, 1, 2, 4, and 6 h), cells were washed twice with PBS and then lysed directly in culture dishes using 1 mL of Trizol (Invitrogen Corp, Carlsbad, CA). Total RNA isolation and extraction were performed using the manufacturer’s protocol (Invitrogen, Carlsbad, CA, United States). Then the RNA was used to detect lncRNA stability by qRT-PCR. And the qRT-PCR primers MEG3-FLAG were used for detecting expression of both haplotypes. In addition, we also used the GAPDH, as a qRT-PCR reference gene, in order to evaluate the efficiency of the assay. These primers (MEG3-FLAG and Primer-GAPDH) were shown in the Supplementary Table S2. The lncRNA secondary structures and free energy of the two MEG3 mutants were extrapolated by nucleic acid (RNA) folding and thermodynamic methods (Zuker, 2003).

### Immunofluorescence Staining

Cells were washed twice with PBS and fixed by incubating with 4% paraformaldehyde for 15 min. The cells were then permeabilized with 0.1% Triton X-100 for 20 min. After blocking with 5% bovine serum albumin (BSA) in PBS for 60 min, the cells were incubated with primary antibody anti-Pax7 (1:200, Santa Cruz Biotechnology, Inc., Europe) at 4°C for 12 h. The cells were washed three times with PBS and incubated with TRITC labeled secondary antibody (1:100, Bioworld, United States) for 60 min according to the manufacturer’s instructions. The cells were placed in 4′, 6-diamidino-2-phenylindole (DAPI) for 10 min after washing three times with PBS. The cells were visualized and photomicrographed under an inverted fluorescence microscope (Nikon, Japan).

### Statistical Analysis

All experiments were performed in triplicate, and a representative experiment was selected for presentation. Data were exhibited as the mean ± SEM. Statistical analyses were conducted using GraphPad Prism 7.0 (GraphPad Software Inc., La Jolla, CA, United States). An independent Chi-square test (χ^2^) was used to analyze the connection between SNPs located in MEG3 and meat quality trait in pig via SPSS v19.0 (IBM, United States). For the gene expression level analysis, the comparisons between two groups were evaluated using LSD *t*-test and the significant differences between more groups were determined by one-way analysis of variance (ANOVA). *P* < 0.05 (^∗^) and *P* < 0.01 (^∗∗^) were considered significantly different.

## Results

### SNP Linkage Disequilibrium and Association With Meat Quality Trait

Direct sequencing of the MEG3 gene was performed to detect potential polymorphisms in lean-type and fat-type pigs. Eight SNPs located in the second exon of MEG3 (g.3087A > G, g.3108A > G, g.3252A > G, g.3397T > C, g.3398A > G, g.3766A > G, g.3785T > A and g.3971T > G) were detected. No SNP was screened in the first exon (Table 1).

**TABLE 1:** Position of eight SNPs in MEG3′ Exon2 of lean-type and fat-type pigs.

Number	SNPs designation	GenBank accession number for the SNPs	^*a*^Single marker association (*P*-value)
1	g.3087C > T	rs81286029	<0.01
2	g.3108C > T	rs344501106	<0.01
3	g.3252C > T	rs81286030	0.0705
4	g.3397A > G	rs702667418	<0.001
5	g.3398C > T	rs325797437	<0.001
6	g.3766C > T	rs345662022	<0.001
7	g.3785A > T	rs334059356	<0.001
8	g.3971A > C	rs322802425	<0.001

*^*a*^The association between the SNPs and meat-type of pigs.*

Genetic diversity analysis was performed to calculate genetic indices (Ho, He, and PIC) in fat-type pigs and lean-type pigs (Table 2). According to the classification of PIC value (PIC value < 0.25, low polymorphism; 0.25 < PIC value < 0.5, intermediate polymorphism; and PIC value > 0.5, high polymorphism) (Cheng et al., 2016a), five SNPs (g.3087C > T, g.3108C > T, g.3398C > T, g.3785A > T, g.3971A > C) of the investigated populations mainly belonged to intermediate and high polymorphism.

**TABLE 2:** Genetic diversity parameters of MEG3 among pig breeds.

SNPs	Type	Number^*a*^	Genotype frequency			Allelic frequency		Ho^*b*^	He^*c*^	PIC^*d*^
g.3087C > T (rs81286029)			CC	CT	TT	C	T			
	lean	85	0.18	0.17	0.65	0.27	0.73	0.83	0.17	0.463
	fat	75	0.20	0.36	0.44	0.38	0.62	0.64	0.36	0.561
g.3108C > T (rs344501106)			CC	CT	TT	C	T			
	lean	85	0.17	0.18	0.65	0.26	0.74	0.82	0.18	0.463
	fat	75	0.19	0.37	0.44	0.38	0.62	0.63	0.37	0.557
g.3252C > T (rs81286030)			CC	CT	TT	C	T			
	lean	85	0.90	0.05	0.05	0.93	0.07	0.95	0.05	0.177
	fat	75	0.96	0.04	0	0.98	0.02	0.96	0.04	0.074
g.3397A > G (rs702667418)			AA	AG	GG	A	G			
	lean	85	0.30	0.40	0.30	0.50	0.50	0.60	0.40	0.587
	fat	75	1.00	0	0	1.00	0	1.00	0	0
g.3398C > T (rs325797437)			CC	CT	TT	C	T			
	lean	85	0.22	0.16	0.62	0.30	0.70	0.84	0.16	0.483
	fat	75	0.85	0.08	0.07	0.89	0.11	0.92	0.08	0.251
g.3766C > T (rs345662022)			CC	CT	TT	C	T			
	lean	85	0.70	0.18	0.12	0.79	0.21	0.82	0.18	0.417
	fat	75	0.87	0.13	0	0.93	0.07	0.87	0.13	0.201
g.3785A > T (rs334059356)			AA	AT	TT	A	T			
	lean	85	0.09	0.09	0.82	0.14	0.86	0.91	0.09	0.289
	fat	75	0.33	0.40	0.27	0.53	0.47	0.60	0.40	0.584
g.3971A > C (rs322802425)			AA	AC	CC	A	C			
	lean	85	0.32	0.22	0.46	0.43	0.57	0.78	0.22	0.564
	fat	75	0.71	0.11	0.18	0.77	0.23	0.89	0.11	0.406

*^*a*^Number: Lean-type pigs: 22 Yorkshire pigs, 23 Duroc pigs, 20 Landrace pigs, 20 Pietrain pigs. Fat-type pigs: 75 BaMa Pigs. ^*b*^Ho, gene homozygosity. ^*c*^He, gene heterozygosity. ^*d*^PIC, polymorphism information content.*

The genotype frequency of each SNP in fat-type and lean-type pigs was analyzed. Seven of the eight SNPs (g.3087C > T, g.3108C > T, g.3397A > G, g.3398C > T, g.3766C > T, g.3785A > T, and g.3971A > C) were significantly associated with meat quality phenotype by using chi-square test (*P* < 0.01, *P* < 0.001) (Table 1). However, the genotypic distribution for g.3397A > G, g.3766C > T, and g.3785A > T deviated from the Hardy–Weinberg equilibrium. Thus, the four SNPs, g.3087C > T, g.3108C > T, g.3398C > T, and g.3971A > C were selected for further LD blot analysis. The results suggested that the four SNPs showed linkage inheritance and formed four haplotypes. The haplotype composed of alleles (CCCA) was the advantageous haplotype in fat-type pigs and had higher frequency (61.2%) than that in lean-type pigs (38.8%) (*P* < 0.001). The frequency of haplotype TTCC was higher in lean-type pigs (71.4%), indicating that TTCC was the advantageous haplotype of lean-type pigs (*P* < 0.001) (Table 3).

**TABLE 3:** Haplotypes frequency of the block and associations with meat quality.

Haplotypes		CCCA	TTCA	TTTC	TTCC
Haplotype frequency	Fat-type pigs	0.612	0.543	0.490	0.286
	Lean-type pigs	0.388	0.457	0.510	0.714
*P*-value		<0.001	0.030	0.292	<0.001

*Haplotypes constructed using the following SNPs, rs81286029, rs344501106, rs325797437, rs3228.*

### SNPs of MEG3 Affected LncRNA Expression Level

SCs were isolated and identified by immunofluorescence with anti-Pax7 monoclonal antibody to demonstrate the effect of four linkage SNPs on MEG3 expression. Pax7, which is the marker of SCs, was detected in all of these cells (Figure 1A). According to the frequency analysis of haplotypes, the frequency of MEG3-CCCA in lean-type pigs was higher than that in fat-type pigs (*P* < 0.05). Meanwhile, the frequency of MEG3-TTCC in fat-type pigs was higher than that in lean-type pigs (*P* < 0.05). Therefore, MEG3-CCCA and MEG3-TTCC were represented for genotype of fat-type pigs and lean-type pigs, respectively. Therefore, MEG3-CCCA and MEG3-TTCC sequences with a flag tag were subcloned into the pcDNA3.1 (+) vector and named pcDNA3.1-MEG3-CCCA and pcDNA3.1-MEG3-TTCC, respectively (Figure 1B). Furthermore, the two expression vectors were transfected into SCs. A pair of primer (MEG3-FLAG) was used to detect MEG3 expression after transfection for 48 h. The reverse primer was designed on the sequence of the FLAG tag to avoid the interference of internal MEG3. Based on the RT-PCR results, MEG3 expression was detected in SCs transfected with pcDNA3.1-MEG3-CCCA and pcDNA3.1-MEG3-TTCC by using this primer (MEG3-FLAG) but not in SCs transfected with the pcDNA 3.1 (+) vector (Figure 1C). The MEG3 expression level of the pcDNA3.1-MEG3-TTCC group was significantly higher than that of the pcDNA3.1-MEG3-CCCA group after transfection for 48 h (*P* < 0.01) (Figure 1D).

### Detection of the Stability and Prediction of the Secondary Structures of MEG3 Haplotypes

**FIGURE 2:**
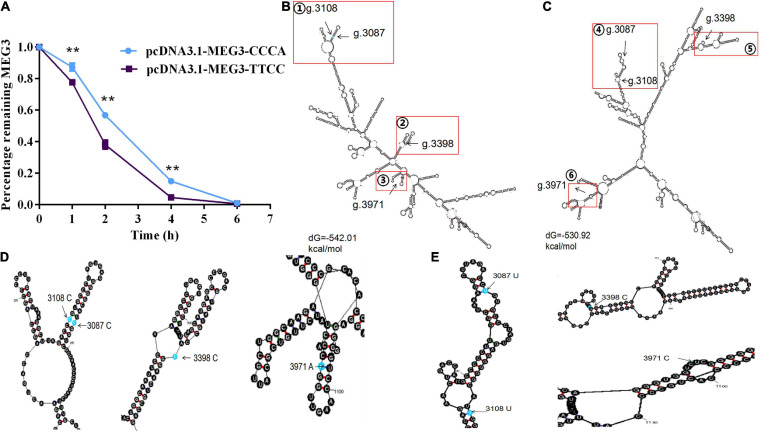
The effect of haplotypes on the stability of MEG3 and the prediction of their secondary structure. **(A)** Percentage of remaining MEG3 level in SCs transfected with pcDNA3.1-MEG3-CCCA or pcDNA3.1-MEG3-TTCC and incubated with 20 μg/mL actinomycin D for appointed time. The significance test: **P < 0.01. **(B,C)** showed secondary structure and minimum free energy of the MEG3-CCCA and MEG3-TTCC, respectively. Four SNP sites were marked with red squares. **(D,E)** showed the enlarged views of the area indicated by the red rectangle from **(B,C)**, respectively.

**FIGURE 3:**
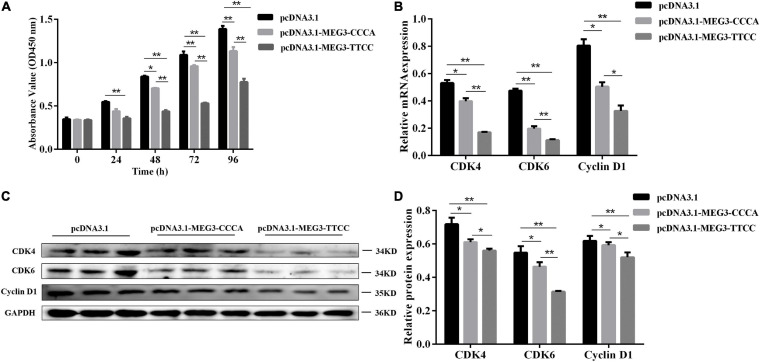
MEG3 haplotypes overexpression suppressed proliferation of SCs. **(A)** Cell proliferation was measured by CCK-8 at 48 h after transfection of pcDNA 3.1 (+), pcDNA3.1-MEG3-CCCA or pcDNA3.1-MEG3-TTCC in SCs. **(B)** The mRNA expression of proliferation-related genes in pcDNA 3.1 (+), pcDNA3.1-MEG3-CCCA or pcDNA3.1-MEG3-TTCC groups by qRT-PCR analysis. **(C,D)** Detection and expression level of proliferation-related protein in pcDNA 3.1 (+), pcDNA3.1- MEG3-CCCA, or pcDNA3.1-MEG3-TTCC groups by western blot assay. The significance test: *P < 0.05, **P < 0.01.

The lncRNA stability test was carried out to determine whether difference in the expression level of MEG3-CCCA and MEG3-TTCC are caused by variations in lncRNA stability. As shown in Figure 3A, the inhibition level of MEG3 transcription was significantly different between two groups by blocking lncRNA transcription with actinomycin D (ActD; 20 μg/mL). The amount of MEG3 in the pcDNA3.1-MEG3-TTCC group decreased faster than that in the pcDNA3.1-MEG3-CCCA group (*P* < 0.05) (Figure 2A). MEG3 secondary structures were determined using Mfold web server given the significant difference in RNA stability between MEG3-TTCC and MEG3-CCCA haplotypes (Figures 2B–E). The predicted structures of the two lncRNA-MEG3 haplotypes differed from each other. Furthermore, the minimal free energy levels of MEG3-CCCA and MEG3-TTCC were −542.01 and −530.92 kcal/mol, respectively.

### MEG3 Haplotypes Overexpression Inhibited SC Proliferation

CCK-8 assay was conducted to understand the potential involvement of MEG3-CCCA and MEG3-TTCC in proliferative capacity. Compared with the pcDNA3.1 (+) group, the pcDNA3.1-MEG3-CCCA group showed no significant effect on cell proliferation at 24 h after transfection. However, at 48 h (*P* < 0.05) as well as 72 and 96 h (*P* < 0.01) after transfection, the pcDNA3.1-MEG3-CCCA group showed inhibited SC proliferative capacity compared with the pcDNA3.1 (+) group. Moreover, cell proliferation in the pcDNA3.1-MEG3-TTCC group was markedly inhibited compared with that in the pcDNA3.1 (+) group at 24, 48, 72, and 96 h (*P* < 0.01). Furthermore, the proliferative capacity in the pcDNA3.1-MEG3-TTCC group sharply decreased compared with that in the pcDNA3.1-MEG3-CCCA group at 48, 72, and 96 h (*P* < 0.01) (Figure 3A). Moreover, the expression levels of genes involved in proliferation including CDK6, CDK4, and CyclinD1 were analyzed (Yang et al., 2007; Wang et al., 2017). The results of qRT-PCR and Western blot assay suggested that the expression levels of CDK4, CDK6, and CyclinD1 in both pcDNA3.1-MEG3-CCCA and p-M-T groups were markedly downregulated compared with those in the pcDNA3.1 (+) group (*P* < 0.05). The expression of all these genes in the pcDNA3.1-MEG3-TTCC group was lower than that in the pcDNA3.1-MEG3-CCCA group (*P* < 0.05) (Figures 3B–D).

### MEG3 Haplotypes Overexpression Promoted SC Differentiation

**FIGURE 4:**
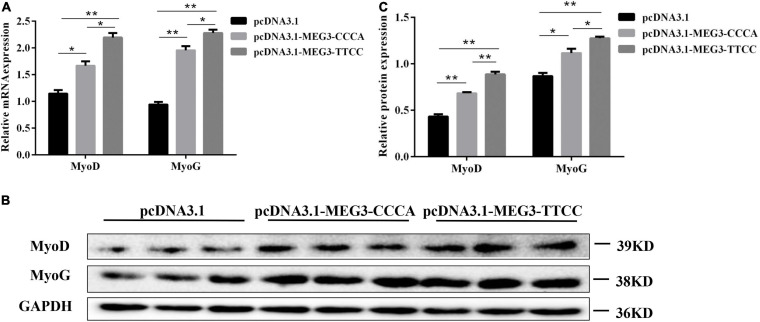
MEG3 haplotypes overexpression promoted differentiation in SCs. **(A)** The mRNA expression of differentiation-related genes in pcDNA 3.1 (+), pcDNA3.1-MEG3-CCCA, or pcDNA3.1-MEG3-TTCC groups by qRT-PCR analysis. **(B,C)** Western blot assay was performed to examine differentiation-related protein expression in pcDNA 3.1 (+), pcDNA3.1-MEG3-CCCA, or pcDNA3.1-MEG3-TTCC groups. The significance test: *P < 0.05, **P < 0.01.

To further determine the effect of MEG3 haplotypes on the differentiation of SCs, we transfected the cells with pcDNA3.1-MEG3-CCCA or pcDNA3.1-MEG3-TTCC. The transfected SCs were cultured in GM for 24 h (Day 0) and induced to cell differentiation in DM. The cells were harvested on day 4 and then analyzed by qRT-PCR and Western blot. The expression levels of two classic myogenic markers, namely, MyoD and MyOG, were analyzed by qRT-PCR and Western blot assay. The overexpression in pcDNA3.1-MEG3-CCCA and pcDNA3.1-MEG3-TTCC groups in SCs led to upregulation of MyoD and MyoG at RNA (Figure 4A) and protein (Figures 4B,C) levels compared with that in the control group (*P* < 0.05). Moreover, the RNA (Figure 4A) and protein (Figures 4B,C) expression levels of MyoD and MyoG in the pcDNA3.1-MEG3-CCCA group were significantly lower than those in the pcDNA3.1-MEG3-TTCC group (*P* < 0.05).

### MEG3 Haplotypes Overexpression Inhibited SC Proliferation by Suppressing PI3K/AKT and MAPK/ERK1/2 Pathways

**FIGURE 5:**
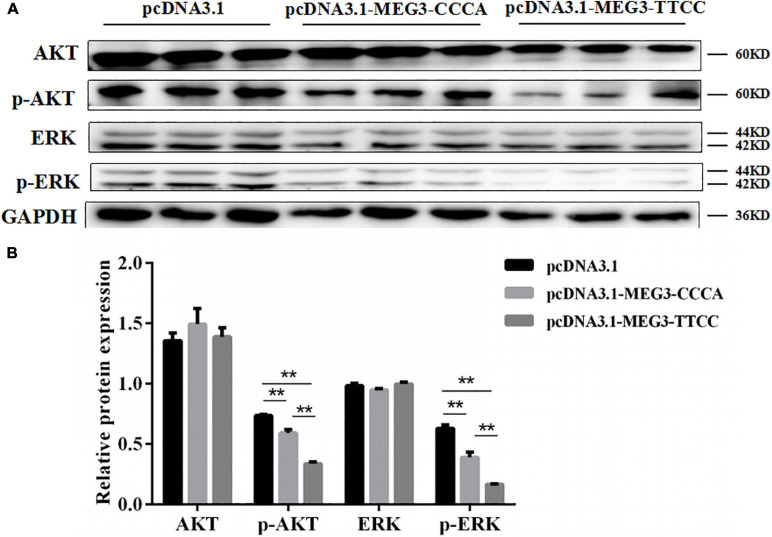
Overexpression of MEG3 haplotypes inhibited PI3K/AKT and MAPK/ERK1/2 signaling pathways in SCs. Western blotting analysis of the expression of AKT, p-AKT, ERK1/2, and p-ERK1/2 **(A)** and their relative expression level **(B)** in pcDNA 3.1 (+), pcDNA3.1-MEG3-CCCA, or pcDNA3.1-MEG3-TTCC groups. The significance test: **P < 0.01.

Western blot analysis was performed to investigate the effects of overexpression of different MEG3 haplotypes on PI3K/AKT and MAPK/ERK1/2 signaling pathways and determine the potential mechanism by which MEG3 haplotypes regulate SC proliferation. The Western blot result showed that MEG3-CCCA and MEG3-TTCC overexpression significantly reduced the levels of phosphorylated AKT and phosphorylated ERK1/2 (*P* < 0.01) (Figures 5A,B). Furthermore, the expression levels of phosphorylated AKT and phosphorylated ERK1/2 in the group transfected with pcDNA3.1-MEG3-TTCC significantly decreased compared with those in the pcDNA3.1-MEG3-CCCA group (*P* < 0.01) (Figures 5A,B).

### MEG3 Haplotypes Overexpression Promoted SC Differentiation by Activating JAK2/STAT3 Pathway

**FIGURE 6:**
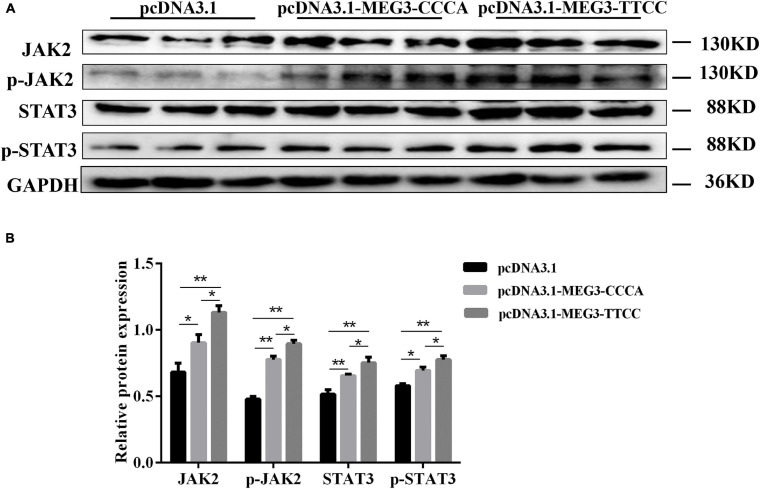
Overexpression of MEG3 haplotypes inactivated JAK2/STAT3 signaling pathway in SCs. **(A)** SCs lysates were immunoblotted with anti-JAK2, p-JAK2, STAT3, and p-STAT3 antibodies in pcDNA 3.1(+), pcDNA3.1-MEG3-CCCA, or pcDNA3.1-MEG3-TTCC groups. **(B)** Relative Protein expressions level of JAK2, p-JAK2, STAT3, and p-STAT3. The significance test: *P < 0.05, **P < 0.01.

To elucidate the possible mechanism of MEG3 haplotypes in regulating SC differentiation, we performed Western blot assay and examined the effects of overexpressing different MEG3 haplotypes on the JAK2/STAT3 signaling pathway. As shown in Figures 6A,B, the Western blot indicated that the protein levels of total and phosphorylated JAK2 and STAT3 increased in pcDNA3.1-MEG3-CCCA and pcDNA3.1-MEG3-TTCC transfection groups (*P* < 0.05). The expression levels of JAK2, p-JAK2, STAT3, and p-STAT3 were significantly enhanced in the pcDNA3.1-MEG3-TTCC group than those in the pcDNA3.1-MEG3-CCCA group (*P* < 0.05) (Figures 6A,B).

## Discussion

LncRNAs have been increasingly implicated to play a crucial role in muscle development and have emerged as significant regulators of muscle proliferation and differentiation (Lim et al., 2018). Previous studies demonstrated the MEG3 affected essential cell processes, such as differentiation, proliferation, and apoptosis (Fu et al., 2018; Xu et al., 2018; Liu et al., 2019). MEG3 also affected momentous cell signaling pathways, including PI3K/AKT, MAPK/ERK, JAK2/STAT3, and p53, in all cell lines (Wang et al., 2008; Fu et al., 2018; Xu et al., 2018).

SNPs, which occur within lncRNAs, affect the structure, minimal free energy, and expression of lncRNA molecules (Minotti et al., 2018). Consequently, SNPs affect the function of lncRNAs (Minotti et al., 2018). Yang et al. (2018) suggested that rs7158663 and rs4081134 influenced the expression levels of MEG3 in lung cancer. The rs4081134 polymorphism was speculated to alter the centroid secondary structure and minimal free energy, which changed the folding of MEG3. The rs941576 located in MEG3 was proved to be significantly associated with type I diabetes, which contributed to skeletal muscle defects and serious atrophy (Tang et al., 2017). In the present study, eight SNPs of MEG3 were detected in different meat-type pigs. All of the SNPs presented median polymorphisms, indicating their large genetic variations and selection potentials. Furthermore, four of the eight SNPs (g.3087C > T, g.3108C > T, g.3398 C > T, and g.3971A > C) were significantly associated with meat quality phenotype (*P* < 0.001). These results suggested that the selection pressure on these four loci in the population was powerful and effective. The haplotype referred to physical arrangement of multiple SNPs on the same inherited chromosome (Cai et al., 2014) and played a crucial role in association studies (Chen et al., 2013). Cai et al. (2014) pointed out that the analysis of haplotype would be more powerful and effective in detecting an association than those of individual SNPs. In the present study, haplotype analysis was performed. The haplotypes of CCCA and TTCC were the advantageous haplotype in fat-type pigs and lean-type pigs, respectively. Increasing studies revealed that different haplotypes affect gene expression and play an important role in diseases (Cheng et al., 2016b; Maslah et al., 2017). Cheng et al. (2016b) revealed that different haplotypes in the signal sequence of GH gene changed the expression of mRNA of GH.

To clarify the function of two haplotypes of MEG3, we constructed their expression vectors and examined their effects on MEG3 expression. The expression level of MEG3-TTCC was higher than that of MEG3-CCCA at the transcription level (*P* < 0.01). Previous studies demonstrated that SNPs could disrupt the stability of lncRNAs and affect their molecular function and expression (Minotti et al., 2018). Whether the differences in the transcription level of MEG3 are due to the changes in MEG3 stability remains unknown. In the present study, actinomycin D was used to treat SCs to determine whether the different transcription levels of MEG3 could be evoked by different levels of lncRNA stability (Seiler et al., 2017). The stability of MEG3-CCCA was higher than that of MEG3-TTCC. At present, Mfold web servers were developed to use dynamic programming to predict the effect of single or multiple-point mutations on the RNA secondary structure by minimum free energy of the molecule, which may reflect the stability of RNA (Sabarinathan et al., 2013). In the present study, we found that MEG3-CCCA was more stable than MEG3-TTCC and that the minimum free energy in the former was −11.09 Kcal/mol higher than that in MEG3-TTCC, consistent with the lncRNA stability detected. Therefore, we speculated that SNPs may change the stability and RNA secondary structure of MEG3 and consequently affect MEG3 transcription.

In our present study, CCK8 assay and detection of proliferation marker genes (CDK4/CDK6/CyclinD1) showed that both MEG3 haplotypes inhibited cell proliferation. This finding illustrated the antiproliferative capacity of MEG3, which was coincided with the former studies (Ji and Li, 2019). Interestingly, the TTCC haplotype significantly decreased the cell proliferative capacity compared with the CCCA haplotype. Besides that, in the present study, we further proved that the two MEG3 haplotypes affected cell differentiation in different degrees among which MEG3-TTCC more significantly promoted differentiation compared with MEG3-CCCA. The possible reason why the two haplotypes had different effects on cells was that SNPs could disrupt the RNA secondary structure of the lncRNAs, influence lncRNA-protein interactions or affect co-expression networks, which affected their molecular function in cells. The relative molecular mechanism needs further experiments to prove.

PI3K/AKT and MAPK/ERK1/2 signaling pathways are widely implicated in regulating myoblast proliferation and development of muscles (Fu et al., 2018; Ji and Li, 2019). MEG3 reduces the growth of glioma cells by inactivating the PI3K/AKT signaling pathway (Zhang et al., 2017). Furthermore, MAPK/ERK1/2 signaling was proved to exert considerable roles in protein synthesis during skeletal muscle cell proliferation (Fu et al., 2018). Our results showed that PI3K/AKT and MAPK/ERK1/2 signaling pathways were inhibited in varying degrees by overexpressing MEG3 haplotypes. The overexpression of MEG3-TTCC showed stronger inhibition on the two signal pathways. Moreover, multiple studies suggested that the JAK2/STAT3 signaling pathway was indispensable for myogenic differentiation by regulating the expression of MyoD and MEF (Wang et al., 2008). Our results suggested that MEG3 mutants activated the JAK2/STAT3 signaling pathway in different degrees. MEG3-TTCC had strong active effect on the JAK2/STAT3 signal pathway. These findings may be due to the changes in the stability and secondary structure of MEG3 caused by SNPs, which finally affected the interaction of MEG3 with target genes. However, the interactions between MEG3 and the target genes in muscle differentiation remain unclear.

Taken together, the four SNPs located in MEG3, which formed two advantageous haplotypes (CCCA and TTCC) in fat-type and lean-type pigs, affected the transcription of MEG3. Overexpression of different MEG3 haplotypes inhibited SC proliferation through the inactivation of PI3K/AKT and MAPK/1/2/ERK pathways. Upregulation of MEG3 haplotypes promoted SC differentiation via the JAK2/STAT3 pathway. This study extended knowledge on the effects of SNPs located in MEG3 during skeletal muscle development and provided novel insight into the biological function of MEG3 in skeletal muscle development. However, the interactions of MEG3 with the target genes in porcine muscle differentiation need to be further investigated in the future.

## Data Availability Statement

All datasets generated for this study are included in the article/Supplementary Material, further inquiries can be directed to the corresponding author/s.

## Ethics Statement

The animal study was reviewed and approved by the Institutional Animal Care and Use Committee of Jilin University.

## Author Contributions

LH, RY, YL, and JM conceived and designed the experiments. RY performed the experiments. LH, CW, SW, YC, RY, TF, GL, XS, and JS assessed the experiments and provided the data analysis. RY wrote the manuscript. All authors read and approved the manuscript.

## Conflict of Interest

The authors declare that the research was conducted in the absence of any commercial or financial relationships that could be construed as a potential conflict of interest.
