# Supplementary material for: Effects and Molecular Mechanism of Single-Nucleotide Polymorphisms of MEG3 on Porcine Skeletal Muscle Development
Source: Front Genet. 2021 Feb 22;12:607910. doi: 10.3389/fgene.2021.607910 (PMC7937967; doi:10.3389/fgene.2021.607910)
Supplement: Supplementary file 1 [file Data_Sheet_1.pdf]

Table S1 Characteristics of different pig breeds

Pig breeds	Average weight (kg)	Lean meat percentage (%)	Average back fat thickness (mm)	Fat percentage (%)	Intramuscular fat (%)	Carcass weight (kg)
Yorkshire	101.83±0.92	64.36±0.17	10.30±0.25	16.47±0.10	2.09±0.08	75.56±0.86
Landrace	96.3±2.34	66.03±0.67	18.60±0.89	11.37±0.28	2.16±0.11	71.0±2.31
Duroc	98.62±1.08	68.24±0.21	12.72±0.40	13.06±0.37	2.99±0.07	72.72±1.06
Pietrain	99.50±1.04	73.14±0.68	16.03±0.86	11.62±0.73	2.47±0.11	74.10±1.15
BaMa	41.59±1.14	45.99±1.22	12.02±0.22	25.1±0.61	6.37±0.46	21.1±0.14

Table S2 Primer pairs used for this work

Primers	Sequences (5' -3')	Product lengths (bp)	Annealing temperature (℃)
MEG3-Exon 1	F 5'-CAAATCGTGCACCGAAATC-3'<br>R 5'-CACTCGTCTTGATGATGCCTTA -3'	670	58
MEG3-Exon 2	F 5'-CCATGCCTTAGGGTCTGTCTT -3'<br>R 5'-AAAGTGGCGGTCAAACGAG -3'	1962	58
MEG3-FLAG	F 5'-CATTTGACAGTGGGACAGC-3'<br>R 5'-GTCATCGTCGTCCTTGTAGTC-3'	203	59
Primer-GAPDH	F 5'-ATGGTGAAGGTCGGAGTGAAC-3'<br>R 5'-CTCGCTCCTGGAAGATGGT-3'	235	59
CDK4	F 5'-TTCGAGCATCCCAATGTTGTC -3'<br>R 5'-GTCTCGATGAACGATGCAGTTG -3'	225	59
CDK6	F 5'-TTGGCTTTGGTGGGTAGTTCT-3'<br>R 5'-TGAATGTCGGGTAGGAAGATTG-3'	322	59
CyclinD1	F 5'-TGCATCTACACCGACAACTCCA -3'<br>R 5'-GTTGGAAATGAACTTCACGTCTGT-3'	222	59
MyoG	F 5'-AGCGCCATCCAGTACATCG -3'<br>R 5'-CACTGTGATGCTGTCCACGAT -3'	261	59
MyoD	F 5'-CGACTTCTATGATGACCCGTGTT-3'<br>R 5'-CGTTAGTGGTCTTGCGTTTGC-3'	242	59
